# Mr. Golding's Case of Parturition

**Published:** 1811-03

**Authors:** Widdows Golding

**Affiliations:** Reading


*To the Editors of the Medical and Physical Journal.*

Gentlemen,

HAVING, in the course of practice, met with an unusual Midwifery case, I am induced to send it for insertion in your Journal, and

Remain, &c. &c.

WIDDOWS GOLDING.

*Reading, Jan.* 31, 1811.

Sept. 25th, 1810.—I was desired to attend M. B. who resides seven miles in the country. I returned with the messenger about eleven A. M. The midwife who was present, informed me she had been with the woman for twenty-four hours. The child, she said, came footling, and had remained in that situation for some hours, as she was unable to get the head away. Upon inquiry, I found the patient was in her forty-first year, and married late in life. Twelve months since, she was delivered of a dead child; the present was her second. She informed me nothing unusual occurred during during the period of gestation. Her pains were now regular, pulse quick, but no hæmorrhage had taken place. On examining the child I found it dead; the midwife had tied a garter round the neck, in which she included the funis, and by the force made use of to bring the head away, all the cervical vertebræ were separated, and the integuments worn so thin by extension, that had I attempted to make use of more force to deliver by the neck, there would have been great danger of separating the head from the body of the child. My first object was to remove the ligature; I then introduced my hand, to examine the situation of the head, and found the chin resting upon the symphysis pubis: it was with more than usual difficulty that the head was brought into its proper position. After waiting a little time to refresh and encourage my patient, I introduced my finger into the mouth of the child to depress the chin, hoping by that means to accelerate the delivery. Meeting with considerable resistance, and knowing the child was dead, I employed more force, at which time the lower jaw separated in the middle. I then passed my fore-finger down the esophagus, and obtained a firm hold, but found the resistance so great that the delivery was impossible. After waiting some time, and finding no advantage had been gained, I suspected some præternatural enlargement of the head. To be perfectly satisfied, I again introduced my hand, and found the body of the uterus completely occupied. I was very particular in this examination, and was convinced that the enlargement was produced by an hydrocephalus internus. With great care I passed up the perforator, and made an opening between the temporal and occipital bones; a profuse discharge of bloody water followed, and soon after the head came away without any difficulty. I waited the usual time, and removed the placenta. I am happy to add, that no unfavourable symptom appeared: the patient soon recovered her strength, and obtained convalescence.

During the delivery, I observed an unnatural appearance on the back of the child, which I now examined more minutely. Upon the lumbar vertebræ, there was a space extending three inches down the spine, and nearly two in breadth, which appeared florid, and the common integuments were wanting. The vertebræ of the spine were all perfect, nor could I discover any aperture that led to that part which I have observed in cases of spina bifida, when situated in the cervical vertebræ.

To ascertain the quantity of fluid contained in the head, I filled it again with water, and it held rather more than two quarts.

